# Comparative Clinical Efficacy of HYBENX® Versus Chlorhexidine Gel as Adjuncts to Scaling and Root Planing: A Split-Mouth Randomized Pilot Clinical Trial

**DOI:** 10.7759/cureus.117602

**Published:** 2026-10-07

**Authors:** Malaz Fayad, Hashem Daoud

**Affiliations:** 1 Department of Periodontology, Latakia University, Latakia, SYR

**Keywords:** adjunctive therapy, chlorhexidine, hybenx, periodontitis, split-mouth design

## Abstract

Background: Scaling and root planing (SRP) has limitations in deep periodontal pockets, necessitating adjunctive therapies. Chlorhexidine (CHX) gel and HYBENX® topical desiccant (EPIEN Medical, Inc., St. Paul, USA) represent chemical antisepsis versus physical desiccation, respectively, but no study has directly compared them in Stage III periodontitis.

Objective: This study aimed to compare the clinical efficacy of HYBENX® versus 1% CHX gel (Hexigel; ICPA Health Products Ltd., Mumbai, India) as adjuncts to SRP in Stage III periodontitis. The primary outcome was the change in probing depth (PD). Secondary outcomes included clinical attachment level (CAL) and bleeding on probing (BOP) over three months.

Methods: This split-mouth, assessor-blinded, randomized trial enrolled 10 patients (100 sites). Following SRP, quadrants received either 1% CHX gel or HYBENX® (60% vinyl polymers, 28% phosphoric acid). The primary outcome was PD, and the sample size was calculated based on this outcome. Secondary outcomes were CAL and BOP. Parameters were recorded at baseline, one month, two months, and three months. Statistical analysis used the Wilcoxon signed-rank test with Bonferroni correction (P = 0.0167).

Results: Both groups improved significantly at one month (P = 0.002). However, the HYBENX® group showed greater improvements at all time points. At three months, the HYBENX® group demonstrated significantly lower BOP (0.38 ± 0.11 vs. 1.32 ± 0.25, P = 0.002) and CAL (3.68 ± 0.29 vs. 4.28 ± 0.44 mm, P = 0.016), while the difference in PD (3.06 ± 0.35 vs. 3.58 ± 0.37 mm, P = 0.027) did not reach statistical significance after Bonferroni correction (adjusted threshold P < 0.0167), despite a large effect size (r = -0.70). The CHX group showed a gradual loss of initial improvement (BOP regression T1-T3, P = 0.004), while the HYBENX® group maintained stable improvements without regression. These findings should be interpreted with caution given the small sample size and short follow-up.

Conclusion: Within the limitations of this pilot trial, HYBENX® showed greater and more sustained clinical improvements compared to CHX gel as an adjunct to SRP in Stage III periodontitis, with stable therapeutic gains over the three-month follow-up. However, given the small sample size and short follow-up, these findings should be considered preliminary and require confirmation in larger, adequately powered trials.

## Introduction

Periodontitis is a chronic inflammatory disease of the periodontium, resulting from a complex interaction between a dysbiotic subgingival microbiota and a dysregulated host immune response [1]. Recent global burden estimates indicate that severe periodontitis affects a substantial proportion of the world's population, with prevalence projected to rise through 2050 [2]. Beyond its oral manifestations, periodontitis has been consistently associated with systemic conditions, including diabetes mellitus and cardiovascular diseases, underscoring its broader public health significance [3].

Non-surgical periodontal therapy, primarily scaling and root planing (SRP), remains the gold standard for the initial management of periodontitis [4]. SRP aims to disrupt and remove the subgingival biofilm and calculus; however, mechanical instrumentation alone has inherent limitations, particularly in deep periodontal pockets (≥6 mm), where complete biofilm removal is frequently unattainable [5]. Consequently, residual pathogens may persist, prompting the search for effective adjunctive therapies to enhance clinical outcomes [6].

Among locally delivered antimicrobials, chlorhexidine (CHX) is considered the gold standard chemical plaque control agent owing to its broad-spectrum activity and substantivity [7]. CHX gel has demonstrated clinical benefits as an adjunct to SRP, with improvements in probing depth (PD) and bleeding on probing (BOP) [8]. Nevertheless, the long-term efficacy of CHX is limited by a well-documented side-effect profile encompassing tooth staining and taste alteration [9]. Furthermore, recent systematic reviews have questioned whether the adjunctive use of CHX provides clinically meaningful benefits over SRP alone over extended follow-up periods [10].

In response to the limitations of conventional chemical antiseptics, physical desiccation agents have emerged as a novel strategy. HYBENX® (EPIEN Medical, Inc., St. Paul, USA) is a topical desiccant composed of vinyl polymers and phosphoric acid, which has received U.S. Food and Drug Administration (FDA) clearance for clinical use [11]. Unlike traditional antimicrobials, HYBENX® operates through a unique physical mechanism termed "desiccation shock," rapidly extracting water from the biofilm matrix and causing structural collapse [12]. This mechanism has been proposed to be associated with reductions in pathogenic bacteria and inflammatory cytokines such as IL-1β and TNF-α in previous clinical studies [13]. However, these findings are based on previous literature and require confirmation in larger, well-controlled trials; they are not directly demonstrated by the present study.

Despite the growing evidence supporting the individual efficacy of both agents, no randomized clinical trial has directly compared them in patients with Stage III periodontitis according to the 2017 classification [14]. Previous investigations evaluated HYBENX® separately against SRP alone or against lasers, but have not provided head-to-head evidence against CHX gel [15,16]. The use of adjunctive therapies in advanced periodontitis remains a subject of ongoing scientific debate regarding their true added value [17].

Therefore, the aim of this split-mouth randomized clinical trial was to compare the clinical efficacy of HYBENX® versus 1% CHX gel (Hexigel; ICPA Health Products Ltd., Mumbai, India) as adjuncts to SRP in patients with Stage III periodontitis over a three-month follow-up period. The primary objective was to compare the change in PD between the two groups, and this outcome was used for sample size calculation. Secondary objectives included comparisons of clinical attachment level (CAL) and BOP changes between the two groups.

## Materials and methods

Study design and participants

This study was designed as a prospective, split-mouth, assessor-blinded, randomized controlled clinical trial conducted at the Department of Periodontology, Faculty of Dentistry, Latakia University, between December 2025 and March 2026. The trial was approved by the Ethics Committee of the Faculty of Dentistry, Latakia University, Syria (approval number 195) on October 21, 2025, and was conducted in accordance with the ethical principles of the Declaration of Helsinki (2013 revision) [18] and the Consolidated Standards of Reporting Trials (CONSORT) statement for randomized clinical trials [19]. The trial was registered with the researchregistry under the identification number researchregistry12012. The primary outcome (PD) and secondary outcomes (CAL and BOP) were defined in the trial protocol prior to patient recruitment, although the public trial registration did not explicitly state this hierarchy.

A total of 10 patients with Stage III periodontitis, according to the 2017 World Workshop classification of periodontal and peri-implant diseases and conditions [14], were enrolled in this trial. Each patient served as their own control through a split-mouth design, with 10 periodontal sites per patient (five sites per side), yielding a total of 100 periodontal sites (50 sites per group).

Inclusion criteria for participation were as follows: age between 30 and 55 years; diagnosis of generalized Stage III periodontitis according to the 2017 classification, with Grade A, B, or C; at least four periodontal sites per quadrant exhibiting PD ≥ 6 mm and CAL ≥ 4 mm; presence of BOP; absence of systemic diseases that may affect periodontal tissues; non-smokers or ex-smokers who had quit smoking at least one year prior to enrollment; good oral hygiene motivation; and willingness to comply with follow-up visits and maintain proper oral hygiene practices throughout the study period.

Exclusion criteria included: current smoking or use of any tobacco products; uncontrolled systemic diseases such as diabetes mellitus, cardiovascular diseases, or immunocompromising conditions; pregnant or lactating female patients; history of periodontal treatment within the preceding six months; use of systemic antibiotics or anti-inflammatory medications within the preceding three months; presence of teeth with endodontic-periodontal lesions, root fractures, or inadequate restorations; and any known allergy to CHX or the components of HYBENX®.

Randomization and intervention

This was an assessor‑blinded (single‑blind) trial. The examiner who performed all clinical measurements and the statistician who analyzed the data were both blinded to the treatment allocation throughout the study. However, due to the nature of the interventions, blinding of the operator (the periodontist performing SRP and applying the adjunctive agents) and blinding of the patients were not feasible, as the two agents have different appearances, application procedures, and application times (60 seconds for CHX gel vs. 30 seconds for HYBENX®). To minimize potential bias, patients were instructed not to discuss their treatment experience with the examiner during follow‑up visits.

Calibration of the examiner was performed prior to the study on five patients not included in the trial. Measurements were repeated after a one‑week interval, with the examiner blinded to the initial readings during the second session. The intraclass correlation coefficient (ICC) for PD, CAL, and BOP measurements was 0.92, 0.89, and 0.94, respectively, indicating excellent intra‑examiner reliability. The calibration process included training on the use of the UNC-15 probe (Hu-Friedy, Chicago, USA) and standardization of probing force (approximately 0.25 N) to ensure consistency across all measurements.

All non-surgical periodontal therapy, including full-mouth SRP, was performed by the same experienced periodontist using ultrasonic and hand instruments (Gracey curettes, Zaffiro, Germany) under local anesthesia when necessary. SRP was performed in a single session for each patient, with a duration of approximately 45-60 minutes, depending on the extent of deposits and the number of teeth present.

Oral hygiene instructions were provided to all patients prior to the start of active treatment, including proper brushing technique (modified Bass technique) and interdental cleaning with dental floss or interdental brushes.

Following completion of SRP, the two quadrants of each patient (right and left) were randomly allocated to either the test or control group using a computer-generated random number sequence. Randomization was performed at the quadrant level, with the right and left quadrants of each patient allocated to different treatment groups. In each quadrant, the five deepest sites with PD ≥ 6 mm at baseline were selected for analysis to ensure consistency across patients. The randomization sequence was generated by an independent statistician using IBM SPSS Statistics software version 26 (IBM Corp., Armonk, USA). Allocation concealment was maintained using sequentially numbered, opaque, sealed envelopes that were opened by the operator only after completion of SRP and immediately before application of the adjunctive agent.

The control group (CHX group) received adjunctive application of a commercially available 1% CHX gel (Hexigel) applied subgingivally into the periodontal pockets using a blunt-tipped cannula (NaviTip, Ultradent Products, Inc., South Jordan, USA). The gel was applied to five periodontal sites per quadrant, with approximately 0.1 mL per site, and left in place for 60 seconds before gentle rinsing with water (Figure 1).

**Figure 1:**
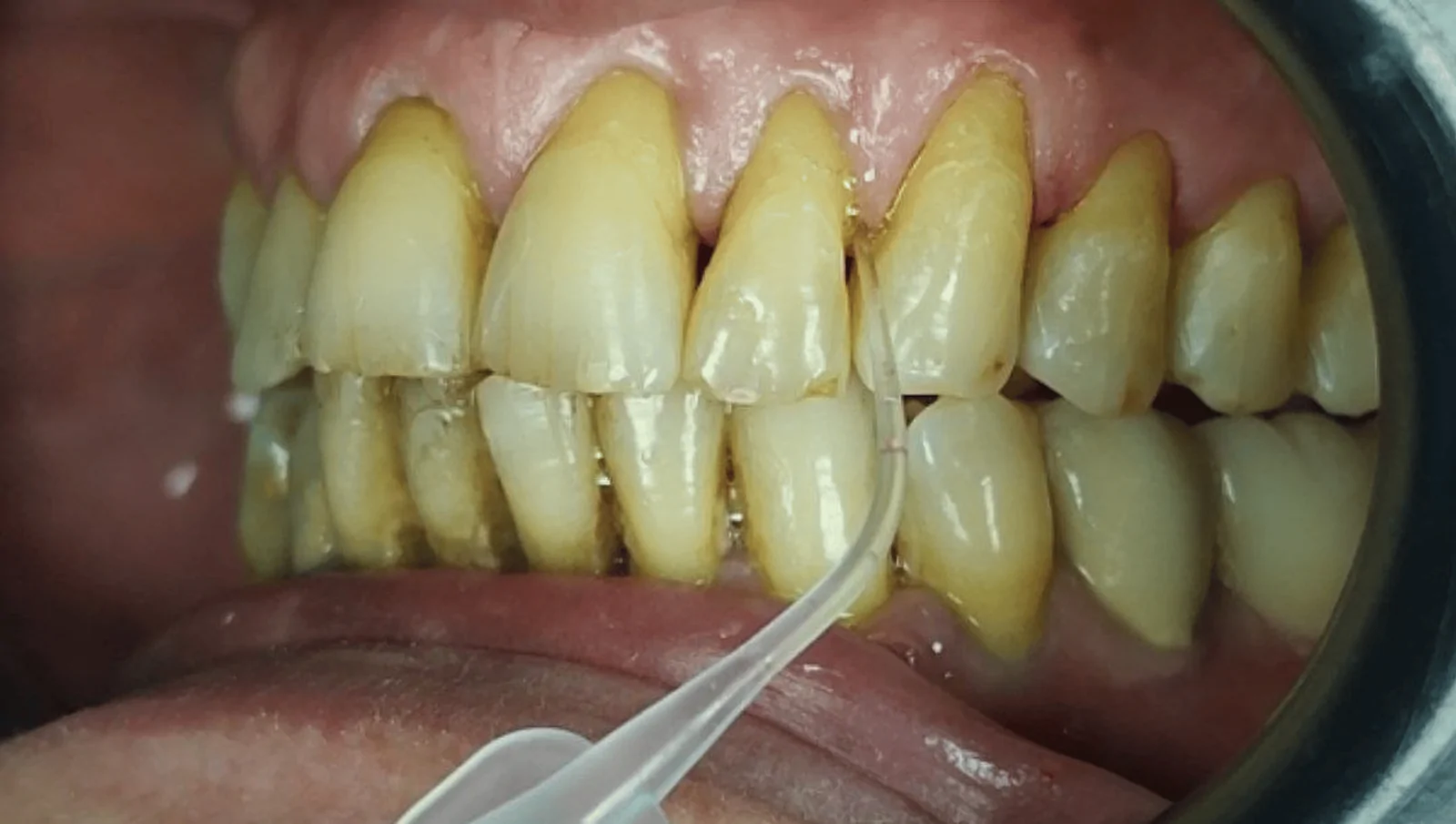
Subgingival application of 1% chlorhexidine (CHX) gel using a blunt-tipped cannula.

The test group (HYBENX® group) received adjunctive application of HYBENX® topical desiccant gel, which contains 60% vinyl polymers and 28% phosphoric acid. HYBENX® operates through a unique physical mechanism termed "desiccation shock," which involves rapid extraction of water from the biofilm matrix, leading to structural collapse and facilitated mechanical removal. The gel was applied subgingivally using a specialized blunt-tipped applicator designed to reach the base of the periodontal pocket, with approximately 0.1 mL per site. The gel was left in contact with the pocket tissues for 30 seconds, after which the area was thoroughly rinsed with water to remove the desiccated biofilm and residual gel (Figure 2).

**Figure 2:**
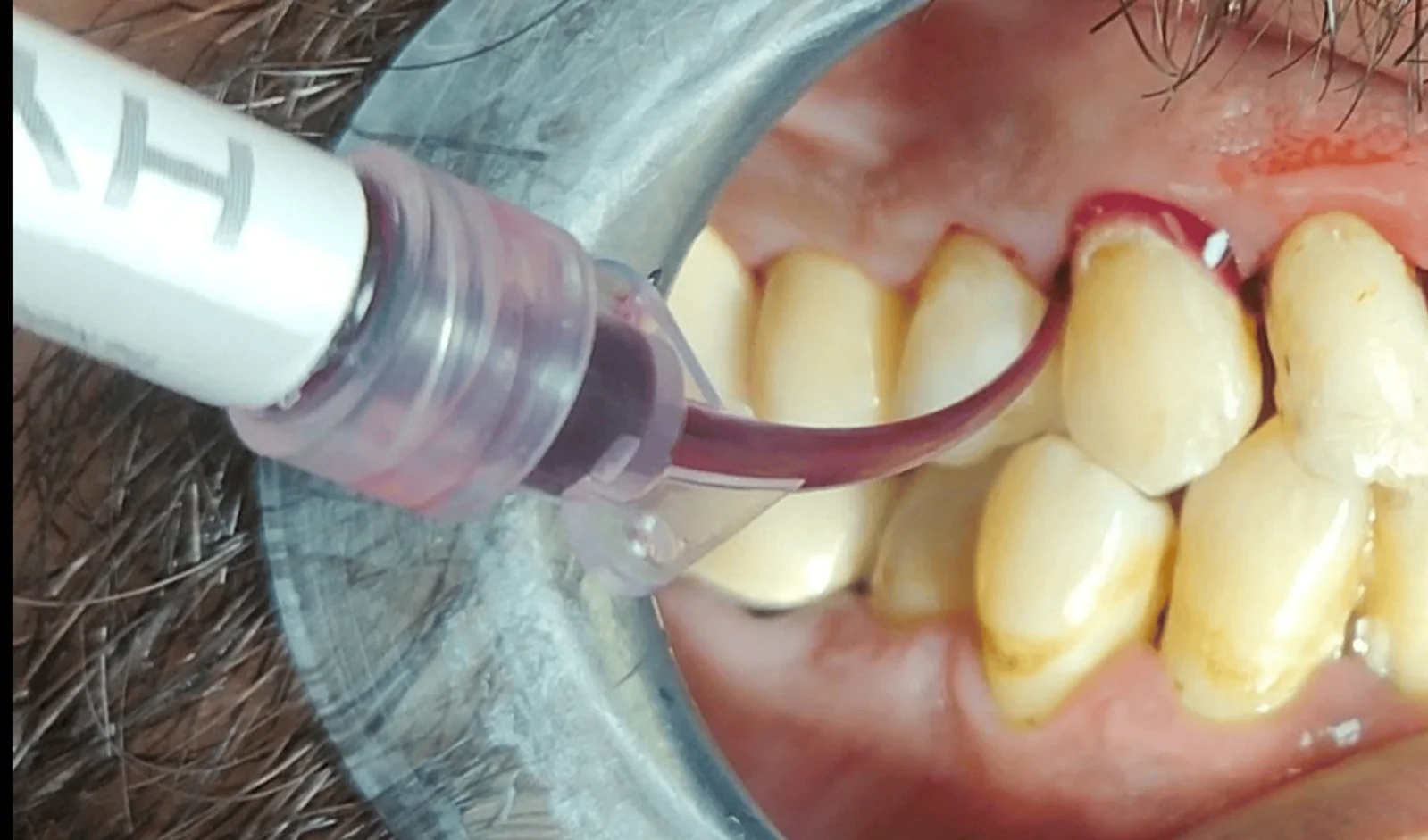
Subgingival application of HYBENX® topical desiccant.

Both adjunctive agents were applied as a single application immediately following SRP. Patients were instructed to avoid eating or drinking for two hours following the procedure and to maintain their regular oral hygiene practices throughout the study period. No adverse events were reported in either group during the study period. Specifically, no tooth staining, taste alteration, mucosal irritation, or discomfort were observed in the CHX group, and no chemical burns, tissue irritation, or discomfort were observed in the HYBENX® group.

Outcome measures

Clinical measurements were recorded at four time points: baseline (T0, prior to SRP), one month (T1), two months (T2), and three months (T3) after treatment. Three clinical parameters were evaluated at six sites per tooth (mesiobuccal, mid-buccal, distobuccal, mesiolingual, mid-lingual, and distolingual), but only sites with PD ≥ 6 mm at baseline were included in the analysis. The inclusion criterion of PD ≥ 6 mm was applied at the site level to ensure inclusion of sites with deep pockets. The baseline mean PD values represent the average of the five selected sites per quadrant, which explains why the mean values are lower than 6 mm. The primary outcome was PD, and the secondary outcomes were CAL and BOP.

Primary Outcome

The primary outcome was the change in PD from baseline to three months. PD was selected as the primary outcome because it is the most direct clinical indicator of periodontal pocket resolution and has been widely used as the primary endpoint in previous adjunctive therapy trials in Stage III periodontitis [6,13]. The sample size calculation was based on PD as the primary outcome. PD was measured as the distance from the gingival margin to the base of the periodontal pocket, using a calibrated UNC-15 periodontal probe with a force of approximately 0.25 N. Measurements were recorded to the nearest millimeter. This hierarchy of outcomes was prespecified in the trial protocol.

Secondary Outcomes

Secondary outcomes included CAL and BOP, which were considered secondary and interpreted with appropriate caution given the multiple comparisons performed.

CAL was measured as the distance from the cementoenamel junction (CEJ) to the base of the periodontal pocket, using the same UNC-15 probe. The CEJ was identified clinically as the distinct anatomic landmark separating the enamel and cementum and was used as a fixed and reproducible reference point for all measurements. The CEJ was used as the reference point for CAL measurements in both the inclusion criteria and all follow-up assessments, ensuring consistency throughout the study. Measurements were recorded to the nearest millimeter. All measurements were performed by the same calibrated examiner using a standardized approach to ensure consistency across all follow-up visits.

BOP was assessed 30 seconds after probing using a manual periodontal probe (UNC-15) at six sites per tooth. Bleeding severity was recorded on a graded (ordinal) scale according to the following criteria: (i) 0 = no bleeding; (ii) 1 = a single bleeding point; (iii) 2 = multiple bleeding points or a fine line; (iv) 3 = bleeding fills the interdental triangle; and (v) 4 = profuse or spontaneous bleeding.

For statistical comparisons and descriptive statistics, the original ordinal scores (0-4) were used. Although BOP was recorded on an ordinal scale (0-4), mean ± standard deviation (SD) values are presented for descriptive purposes to facilitate comparison with previous studies. Non-parametric tests (Wilcoxon signed-rank test) were used for all statistical comparisons, consistent with the ordinal nature of the data. For intra-examiner reliability assessment, scores were dichotomized (0 = no bleeding; ≥1 = bleeding present) to calculate Cohen's kappa coefficient. The reliability analysis was performed separately from the outcome analysis to avoid any confusion between the two.

All clinical measurements were performed by the same calibrated examiner who was blinded to the treatment allocation. To ensure consistency, measurements were taken at the same time of day (morning hours) for each patient across all visits. All sites were probed at each visit, including sites with inflammation, to ensure accurate assessment of BOP. However, sites that had been recently instrumented (within the previous 24 hours) were avoided to prevent false-positive bleeding.

Intra‑examiner reliability was assessed using ICC for continuous variables (PD and CAL). For BOP, although recorded on an ordinal scale (0-4), the scores were dichotomized (0 = no bleeding; ≥1 = bleeding present) for reliability analysis, and Cohen's kappa coefficient was calculated to assess agreement on the presence or absence of bleeding. The kappa value was 0.94, indicating excellent intra‑examiner agreement. All statistical comparisons and descriptive statistics for BOP were performed on the original ordinal scores.

Statistical analysis

Data were analyzed using IBM SPSS Statistics version 26. The patient was the unit of analysis to avoid inflating the sample size and to account for the non-independence of sites within the same patient. Site-level data were aggregated by calculating the mean of the five selected sites per quadrant for each patient, yielding one value per patient per side per parameter (BOP, CAL, and PD) at each time point (n = 10 per group). This approach is consistent with the split-mouth design, where each patient serves as their own control, and provides a more conservative and valid statistical analysis.

An a priori sample size calculation was performed using G*Power software version 3.1 (Heinrich-Heine-Universität Düsseldorf, Düsseldorf, Germany) based on the primary outcome (PD), using a paired t-test, two-tailed, effect size d = 1.0, α = 0.05, and power = 0.80, which indicated a minimum of 10 patients. The paired t-test was used as an approximation for sample size calculation, given the absence of a non-parametric equivalent in G*Power; this approach is conservative and ensures adequate power for the non-parametric analysis. The split-mouth design was chosen to increase statistical efficiency by eliminating inter-individual variability.

The Shapiro-Wilk test showed that most variables deviated from normality (P < 0.05); therefore, non-parametric tests were used throughout. Intra-group and inter-group comparisons were performed using the Wilcoxon signed-rank test. To control for multiple comparisons across the three follow-up time points, the Bonferroni correction was applied to the inter-group comparisons, with the significance level adjusted to P = 0.0167 (0.05 ÷ 3). For intra-group comparisons between consecutive time points (T0-T1, T1-T2, T2-T3), the same adjusted threshold was applied. The primary outcome (PD) was analyzed first, followed by the secondary outcomes (CAL and BOP).

Effect sizes (r) for the Wilcoxon tests were calculated as $r = \frac{Z}{\sqrt{N}}$. 

According to Cohen's criteria, r ≥ 0.50 was considered a large effect. Additionally, 95% confidence intervals (CIs) for the mean differences between groups at each time point were calculated using the paired t-distribution as an approximation, given the absence of a non-parametric equivalent for CI calculation. This approach provides a measure of precision for the treatment effects and is consistent with previous studies in the field. However, this is acknowledged as a potential limitation, and future studies may consider using bootstrap or Hodges-Lehmann CIs for non-parametric data.

Descriptive statistics were presented as mean ± SD. All tests were two-tailed, with statistical significance set at P < 0.05. All Z-values, P-values, effect sizes, and 95% CIs were rechecked against the raw dataset and confirmed to be accurate.

## Results

Demographic characteristics

A total of 10 patients with Stage III periodontitis were enrolled in this split-mouth randomized clinical trial. The sample was equally distributed by sex, with five male patients (50%) and five female patients (50%). Regarding the grade of periodontitis, three patients (30%) were Grade A, five patients (50%) were Grade B, and two patients (20%) were Grade C.

The mean age of participants was 42 ± 5.38 years (range: 35-51 years). Male patients had a mean age of 40 ± 5.87 years, while female patients had a mean age of 44 ± 4.53 years. Each patient contributed 10 periodontal sites (five sites per side), yielding a total of 100 sites, with 50 sites allocated to the CHX group and 50 sites allocated to the HYBENX® group. The demographic characteristics of the study population are summarized in Table 1.

**Table 1:** Demographic characteristics of the study population. SD: standard deviation; CHX: chlorhexidine

Variable	Value
Total number of patients	10
Total number of sites	100
Sex distribution (male / female)	5 (50%) / 5 (50%)
Mean age (years) ± SD	42 ± 5.38
Age range (years)	35-51
Mean age of male patients (years) ± SD	40 ± 5.87
Mean age of female patients (years) ± SD	44 ± 4.53
Sites per group (CHX / HYBENX®)	50 / 50

Descriptive statistics and baseline comparability

Table 2 presents the descriptive statistics for the three clinical parameters (BOP, CAL, and PD) across the four time points in both study groups. At baseline (T0), no statistically significant differences were observed between the two groups for any of the three clinical parameters (BOP: P > 0.05; CAL: P > 0.05; PD: P > 0.05), confirming baseline comparability and validating the split-mouth design.

**Table 2:** Descriptive statistics for clinical parameters across the four time points (mean ± SD). SD: standard deviation; CHX: chlorhexidine; BOP: bleeding on probing; CAL: clinical attachment level; PD: probing depth; T0: baseline; T1: one month; T2: two months; T3: three months

Parameter	Group	T0	T1	T2	T3
BOP	CHX	1.64 ± 0.08	0.82 ± 0.11	1.26 ± 0.33	1.32 ± 0.25
BOP	HYBENX®	1.62 ± 0.11	0.42 ± 0.11	0.48 ± 0.10	0.38 ± 0.11
CAL (mm)	CHX	4.74 ± 0.19	4.08 ± 0.17	4.36 ± 0.31	4.28 ± 0.44
CAL (mm)	HYBENX®	4.64 ± 0.16	3.66 ± 0.16	3.60 ± 0.30	3.68 ± 0.29
PD (mm)	CHX	4.30 ± 0.14	3.62 ± 0.22	3.76 ± 0.25	3.58 ± 0.37
PD (mm)	HYBENX®	4.32 ± 0.14	3.30 ± 0.19	3.02 ± 0.33	3.06 ± 0.35

Primary outcome (PD)

In the CHX group, PD decreased from 4.30 ± 0.14 mm at baseline to 3.62 ± 0.22 mm at T1, then fluctuated to 3.76 ± 0.25 mm at T2 and 3.58 ± 0.37 mm at T3. In the HYBENX® group, PD decreased from 4.32 ± 0.14 mm at baseline to 3.30 ± 0.19 mm at T1, and continued to decrease to 3.02 ± 0.33 mm at T2 and 3.06 ± 0.35 mm at T3. Inter-group comparison showed significant superiority of HYBENX® at T2 (P = 0.002), while the differences at T1 (P = 0.031) and T3 (P = 0.027) did not reach statistical significance after Bonferroni correction (adjusted threshold P < 0.0167). Despite the lack of statistical significance at T1 and T3, the effect sizes were large (r = -0.68 and r = -0.70, respectively), indicating clinically meaningful improvements.

Secondary outcomes (CAL and BOP)

In the CHX group, CAL decreased from 4.74 ± 0.19 mm at baseline to 4.08 ± 0.17 mm at T1, then regressed to 4.36 ± 0.31 mm at T2 and 4.28 ± 0.44 mm at T3. BOP decreased from 1.64 ± 0.08 at baseline to 0.82 ± 0.11 at T1, then increased to 1.26 ± 0.33 at T2 and 1.32 ± 0.25 at T3. In the HYBENX® group, CAL decreased from 4.64 ± 0.16 mm at baseline to 3.66 ± 0.16 mm at T1, and remained stable at 3.60 ± 0.30 mm at T2 and 3.68 ± 0.29 mm at T3. BOP decreased from 1.62 ± 0.11 at baseline to 0.42 ± 0.11 at T1, and remained stable at 0.48 ± 0.10 at T2 and 0.38 ± 0.11 at T3. Inter-group comparisons showed significant superiority of HYBENX® for both secondary outcomes at all follow-up time points (P < 0.05).

Intra-group comparisons

Within the CHX group, all three parameters showed statistically significant improvements at T1 compared to baseline (BOP: P = 0.002; CAL: P = 0.002; PD: P = 0.002). At T2, the improvements remained statistically significant for CAL (P = 0.008) and PD (P = 0.002), while BOP showed a significant difference from baseline (P = 0.008) despite the observed regression. At T3, PD remained significantly improved compared to baseline (P = 0.002), BOP showed a significant difference (P = 0.008), but CAL did not reach statistical significance (P = 0.055), indicating a loss of the initial therapeutic gain for this parameter over time.

In contrast, the HYBENX® group demonstrated highly significant improvements for all three parameters at T1 compared to baseline (all P = 0.002). This improvement was sustained at T2 (all P = 0.002) and T3 (all P = 0.002), with all parameters maintaining highly significant differences from baseline throughout the entire follow-up period. Unlike the CHX group, no regression or loss of statistical significance was observed at any time point, reflecting a stable and sustained therapeutic effect.

Inter-group comparisons at follow-up time points

Table 3 presents the inter-group comparisons at T1, T2, and T3. At T1 (one month), the HYBENX® group showed statistically significant superiority over the CHX group for BOP (P = 0.002) and CAL (P = 0.004), while the difference in PD (P = 0.031) did not reach statistical significance after Bonferroni correction (adjusted threshold P < 0.0167). At T2 (two months), the differences between the two groups widened and remained highly significant for all parameters (BOP: P = 0.002; CAL: P = 0.002; PD: P = 0.002). At T3 (three months), the HYBENX® group continued to demonstrate statistically significant superiority for BOP (P = 0.002) and CAL (P = 0.016), while the difference in PD (P = 0.027) did not reach statistical significance after Bonferroni correction.

**Table 3:** Inter-group comparisons at follow-up time points (Wilcoxon signed-rank test). S: statistically significant (P < 0.0167 after Bonferroni correction); NS: not statistically significant; SD: standard deviation; CHX: chlorhexidine; BOP: bleeding on probing; CAL: clinical attachment level; PD: probing depth; CI: confidence interval; T1: one month; T2: two months; T3: three months

Time point	Parameter	CHX (mean ± SD)	HYBENX® (mean ± SD)	Z value	P value	Effect size (r)	95% CI of difference	Significance
T1	BOP	0.82 ± 0.11	0.42 ± 0.11	-3.09	0.002	-0.98	(0.25, 0.55)	S
T1	CAL (mm)	4.08 ± 0.17	3.66 ± 0.16	-2.89	0.004	-0.91	(0.15, 0.69)	S
T1	PD (mm)	3.62 ± 0.22	3.30 ± 0.19	-2.15	0.031	-0.68	(0.03, 0.61)	NS
T2	BOP	1.26 ± 0.33	0.48 ± 0.10	-3.09	0.002	-0.98	(0.45, 1.11)	S
T2	CAL (mm)	4.36 ± 0.31	3.60 ± 0.30	-3.09	0.002	-0.98	(0.43, 1.09)	S
T2	PD (mm)	3.76 ± 0.25	3.02 ± 0.33	-3.09	0.002	-0.98	(0.41, 1.07)	S
T3	BOP	1.32 ± 0.25	0.38 ± 0.11	-3.09	0.002	-0.98	(0.62, 1.26)	S
T3	CAL (mm)	4.28 ± 0.44	3.68 ± 0.29	-2.42	0.016	-0.77	(0.12, 1.08)	S
T3	PD (mm)	3.58 ± 0.37	3.06 ± 0.35	-2.21	0.027	-0.70	(0.06, 0.98)	NS

The effect sizes (r) for all between-group comparisons ranged from -0.68 to -0.98, indicating large to very large treatment effects. The 95% CI for all comparisons did not cross zero, confirming the statistical significance and clinical importance of the observed differences (Table 3).

Longitudinal changes over time

Table 4 presents pairwise longitudinal comparisons between follow-up time points within each group, including both consecutive (T1-T2, T2-T3) and non-consecutive (T1-T3) comparisons, to assess the stability of the therapeutic effect over time.

**Table 4:** Pairwise comparisons between consecutive follow-up time points within each group (Wilcoxon signed-rank test with Bonferroni correction). NS: not statistically significant; CHX: chlorhexidine; BOP: bleeding on probing; CAL: clinical attachment level; PD: probing depth; T1: one month; T2: two months; T3: three months

Parameter	Group	Comparison	Z value	P value	Significance
BOP	CHX	T1-T2	-2.45	0.018	NS
BOP	CHX	T1-T3	-3.12	0.004	Significant regression
BOP	CHX	T2-T3	-0.42	0.20	NS
BOP	HYBENX®	T1-T2	-0.84	0.25	NS
BOP	HYBENX®	T1-T3	-0.67	0.63	NS
BOP	HYBENX®	T2-T3	-1.21	0.13	NS
CAL (mm)	CHX	T1-T2	-2.15	0.05	NS
CAL (mm)	CHX	T1-T3	-1.45	0.26	NS
CAL (mm)	CHX	T2-T3	-0.78	0.46	NS
CAL (mm)	HYBENX®	T1-T2	-0.92	0.45	NS
CAL (mm)	HYBENX®	T1-T3	-0.42	1.00	NS
CAL (mm)	HYBENX®	T2-T3	-0.67	0.62	NS
PD (mm)	CHX	T1-T2	-1.45	0.20	NS
PD (mm)	CHX	T1-T3	-0.42	0.72	NS
PD (mm)	CHX	T2-T3	-1.21	0.15	NS
PD (mm)	HYBENX®	T1-T2	-3.12	0.004	Sustained improvement
PD (mm)	HYBENX®	T1-T3	-1.45	0.19	NS
PD (mm)	HYBENX®	T2-T3	-0.17	0.88	NS

In the CHX group, a statistically significant regression was observed for BOP between T1 and T3 (P = 0.004), indicating a loss of the initial improvement in bleeding control. For PD, a significant improvement was observed between T1 and T2 (P = 0.004), reflecting a delayed therapeutic effect, while no significant changes were observed between T1 and T3 or between T2 and T3. For CAL, no significant changes were observed between any consecutive time points.

In contrast, the HYBENX® group showed no statistically significant regression for any parameter between any consecutive time points. For PD, a significant and sustained improvement was observed between T1 and T2 (P = 0.004), indicating continued therapeutic effect during the second month, while no significant changes were observed between T1 and T3 or between T2 and T3, reflecting stabilization of the therapeutic gain. For BOP and CAL, no significant changes were observed between any consecutive time points, confirming the stability of the therapeutic effect throughout the follow-up period.

## Discussion

The present split-mouth randomized clinical trial demonstrated that while both adjunctive therapies significantly improved the clinical parameters compared to baseline, HYBENX® exhibited a markedly superior and more sustained therapeutic effect over the three-month period. It should be noted that sites with PD ≥ 6 mm were included in accordance with the 2017 classification for Stage III periodontitis [14]. Non-surgical therapy with adjunctive local agents was chosen as the initial treatment approach, consistent with current clinical guidelines that recommend non-surgical therapy as first-line treatment for Stage III periodontitis [4,17]. Surgical intervention may be required for residual pockets after re-evaluation, and this should be considered in the overall treatment plan.

Regarding the primary outcome (PD), HYBENX® was significantly superior to CHX gel at two months (3.02 ± 0.33 mm vs. 3.76 ± 0.25 mm, P = 0.002), while the differences at one month (P = 0.031) and three months (P = 0.027) did not reach statistical significance after Bonferroni correction (adjusted threshold P < 0.0167). However, the effect sizes at these time points were large (r = -0.68 and r = -0.70, respectively), suggesting that the lack of statistical significance may reflect the small sample size rather than the absence of a clinically meaningful difference. Regarding secondary outcomes, the HYBENX® group also showed significantly greater improvements in CAL (3.68 ± 0.29 mm vs. 4.28 ± 0.44 mm, P = 0.016) and BOP (0.38 ± 0.11 vs. 1.32 ± 0.25, P = 0.002) at three months. These findings should be interpreted with caution, as statistical significance does not always equate to clinical significance, and the small sample size limits the precision of these estimates.

The evaluation of CAL, measured from the CEJ to the base of the periodontal pocket, reflects the true destruction of periodontal supporting tissues and the level of healing, further emphasizing the superiority of the physical desiccant in achieving stable attachment gains [20].

The evaluation of BOP, a critical indicator of active gingival inflammation, revealed distinct patterns between the two groups [21]. In the CHX group, BOP significantly decreased at one month but progressively increased thereafter. Conversely, the HYBENX® group exhibited a sharp and sustained reduction. These findings align with Lombardo et al., who reported sustained reductions in BOP following HYBENX® application alongside ultrasonic debridement [22]. The loss of initial improvement observed in the CHX group can be attributed to the inactivation of the drug by serum proteins and organic debris within deep pockets, as well as bacterial recolonization once the drug concentration diminishes.

Regarding PD, the HYBENX® group demonstrated a continuous and stable reduction, whereas the CHX group showed fluctuating values. The superior efficacy of HYBENX® is consistent with a meta-analysis by Zhao et al., which noted that CHX was less effective than other adjunctive agents in improving PD over time [23]. Furthermore, Pardo et al. confirmed the microbiological and clinical benefits of HYBENX® in reducing pocket depths, supporting our findings of sustained therapeutic gains [24].

The assessment of CAL highlighted that while the CHX group lost its statistical significance by the third month, the HYBENX® group maintained highly significant improvements. This is in agreement with Jain et al., who observed CAL improvements with CHX gel, but the magnitude of gain was notably lower and less stable compared to the desiccation agent [25]. The clinical improvements observed with HYBENX® may be attributed to its proposed mechanism of action, which involves desiccation of the biofilm matrix and facilitation of mechanical removal. However, this mechanistic explanation remains a hypothesis supported by previous literature rather than a finding demonstrated by the present trial [12,13]. Further microbiological and histological studies are needed to confirm these mechanisms.

From a pathophysiological perspective, the periodontal biofilm is composed of approximately 70% water and 10-30% polymeric materials within the extracellular matrix [26]. HYBENX®, being a highly hydrophilic desiccant, is hypothesized to rapidly extract this water, potentially leading to the collapse of the biofilm architecture. Because this mechanism does not rely on direct bactericidal chemical interactions, it may remain effective even in the presence of organic matter, unlike CHX, whose chemical activity is frequently compromised by inflammatory exudates [27].

Furthermore, the side-effect profile of CHX, which includes tooth staining and mucosal irritation, often limits patient compliance during prolonged use [28]. HYBENX®, applied as a single, short-duration procedure, avoids these adverse effects, offering a more patient-friendly alternative.

This study possesses several notable strengths. It is the first randomized clinical trial to directly compare HYBENX® and CHX gel in Stage III periodontitis according to the 2017 World Workshop classification [29]. The split-mouth design effectively eliminated inter-individual variability. In vitro studies support the antimicrobial efficacy of HYBENX® against multidrug-resistant bacteria without inducing cellular toxicity [30]. However, these findings are derived from in vitro models and may not fully reflect the complex in vivo environment. Importantly, this study should be considered exploratory/pilot in nature, given the small sample size and short follow-up. The results should therefore be interpreted as preliminary and hypothesis-generating rather than definitive, and they require confirmation in larger, adequately powered randomized controlled trials with longer follow-up periods.

However, several limitations must be acknowledged. The relatively small sample size and the three-month follow-up period may not capture long-term relapse, as the true therapeutic endpoint of mechanical therapy often requires longer evaluation periods [31]. Additionally, the split-mouth design, while advantageous in reducing inter-individual variability, has inherent limitations, including the potential for carry-over effects between quadrants and the inability to blind patients and operators, which may introduce bias. Furthermore, the small, highly selected sample (non-smokers, systemically healthy, Stage III only) limits the generalizability of the findings to the broader population of periodontitis patients, including smokers, patients with systemic diseases such as diabetes, and those with other stages of periodontitis. The absence of microbiological and cytokine analyses is a significant limitation, as it limits the ability to correlate clinical findings with specific shifts in the subgingival microbiome. Future studies should incorporate microbiological assessment (e.g., PCR or culture) and cytokine analysis (e.g., IL-1β and TNF-α) to provide a more comprehensive understanding of the mechanisms underlying the observed clinical improvements.

Furthermore, although PD was the prespecified primary outcome used for sample size calculation, the trial registration did not explicitly state the hierarchy of outcomes; this has been clarified in the present manuscript. The secondary outcomes (CAL and BOP) were interpreted with caution given the multiple comparisons performed. Additionally, despite the application of Bonferroni correction, the multiple statistical comparisons across three outcomes and several time points may still increase the risk of Type I error, and this should be considered when interpreting the results. Moreover, due to the different appearances, application procedures, and application times of the two agents, blinding of patients and operators was not feasible, which may introduce potential bias. Finally, while CHX gel proved effective initially, its formulation differences compared to CHX solutions might influence substantivity, warranting further investigation into various concentrations and carriers [32]. Future studies with larger sample sizes and longer follow-up periods are warranted to validate these findings.

## Conclusions

Within the limitations of this pilot split-mouth randomized clinical trial, both HYBENX® and CHX gel are effective adjuncts to SRP in Stage III periodontitis. HYBENX® showed greater and more sustained clinical improvements, particularly for CAL and BOP, while maintaining stable therapeutic gains without regression. However, given the small sample size and short follow-up, these findings should be considered preliminary and exploratory. HYBENX® represents a promising adjunctive modality, but further validation in larger, adequately powered studies with longer follow-up is essential before definitive clinical recommendations can be made.
